# A mental models approach for defining explainable artificial intelligence

**DOI:** 10.1186/s12911-021-01703-7

**Published:** 2021-12-09

**Authors:** Michael Merry, Pat Riddle, Jim Warren

**Affiliations:** School of Computer Science, University of Auckland, Symonds St, Auckland, New Zealand

**Keywords:** explainability, xAI, black-box models, mental models

## Abstract

**Background:**

Wide-ranging concerns exist regarding the use of black-box modelling methods in sensitive contexts such as healthcare. Despite performance gains and hype, uptake of artificial intelligence (AI) is hindered by these concerns. Explainable AI is thought to help alleviate these concerns. However, existing definitions for *explainable* are not forming a solid foundation for this work.

**Methods:**

We critique recent reviews on the literature regarding: the agency of an AI within a team; mental models, especially as they apply to healthcare, and the practical aspects of their elicitation; and existing and current definitions of explainability, especially from the perspective of AI researchers. On the basis of this literature, we create a new definition of *explainable*, and supporting terms, providing definitions that can be objectively evaluated. Finally, we apply the new definition of explainable to three existing models, demonstrating how it can apply to previous research, and providing guidance for future research on the basis of this definition.

**Results:**

Existing definitions of explanation are premised on global applicability and don’t address the question ‘understandable *by whom*?’. Eliciting mental models can be likened to creating explainable AI if one considers the AI as a member of a team. On this basis, we define explainability in terms of the *context* of the model, comprising the *purpose*, *audience*, and *language* of the model and explanation. As examples, this definition is applied to regression models, neural nets, and human mental models in operating-room teams.

**Conclusions:**

Existing definitions of explanation have limitations for ensuring that the concerns for practical applications are resolved. Defining explainability in terms of the context of their application forces evaluations to be aligned with the practical goals of the model. Further, it will allow researchers to explicitly distinguish between explanations for technical and lay audiences, allowing different evaluations to be applied to each.

## Background

The use of Artificial Intelligence (AI) models in healthcare holds a lot of promise. However, the use of such algorithms in making decisions regarding sensitive aspects of our lives raises concerns [1]. Black box models, those where we do not understand their inner workings, are of greatest concern. This has been reflected in the General Data Protection Regulation (GDPR) which established the ‘right [...] to obtain an explanation’ of such sensitive decisions [2]. Despite significant work in the area, adoption of such models in practical applications such as clinical decisions support systems has been challenging [3].

Explainable AI (XAI) is new [4], but the foundation of the research goes back to the mid 1970s with work on the explanation of decision support systems [5] with similar motivations to today. Despite work to date, the ongoing concerns indicate a potential mismatch between the work being done and the goals of the AI applications. Many definitions of explanations exist [6–12] but these often start from difficulties in AI research, rather than from the goal. The definitions are not necessarily satisfactory for researchers in other fields [13].

Our objective is to create a new definition of explainability that provides a framework for future work in Explainable AI. This definition is based on the ideas that an AI can be considered a member of a team with agency [14] and that mental models provide a framework for understanding the thinking of members within a team [15]. The rest of the paper addresses the methods to arrive at this point, a review of the relevant literature, the definition itself, worked examples for existing literature, and finally a discussion of the implications of the new definition. This work provides practical steps towards the multidisciplinary approaches to explanation called for by Amann et al. [13].

## Methods

We critique recent reviews on the literature regarding: the agency of an AI within a team; mental models, especially as they apply to healthcare, and the practical aspects of their elicitation; and existing and current definitions of explainability, especially from the perspective of AI researchers. On the basis of this literature, we create a new definition of *explainable*, and supporting terms, providing definitions that can be objectively evaluated. Finally, we apply the new definition of explainable to three existing models, demonstrating how it can apply to previous research, and providing guidance for future research on the basis of this definition.

## Results

The results are presented in three subsections, forming the justification for the new definition of explainability, and the definition itself. The first section establishes that it is possible to consider an AI as an agent or member of a team. By allowing this possibility, it is possible to approach the question of explainability from a different perspective. The second section examines how one elicits explanations from team members when we consider only human team members, and particularly looks at the framework of mental models in which the appropriate process for the elicitation of mental models depends on context. The third section establishes that existing definitions are not well formalised, and that they implicitly rely on a contextual understanding which necessarily differs between applications, causing a tension between a general definition and specific implementation. We argue that if we allow that an AI can be considered as a team member with agency, then mental models, being an accepted framework for explaining thought processes between team members, can be used to resolve the concerns in the definitions of explainability for AI. On that basis, we construct a new definition of explainability, with the new requirement of a well-defined context being necessary for evaluation.

### Healthcare delivery as teamwork (with AIs)

Some consider the output of an AI purely as the output of a statistical model. Clinicians should regard the prediction with appropriate levels of scepticism and uncertainty [16]. The AI and the physician should work together. The model predicts, and the physician explains and acts upon that information [17]. The lack of explanation or comprehension of the model is a concern, thought to be mitigated by the physician’s ability to provide the human context [17]. For this use, further training in statistical methods is recommended for the physicians to be able to interpret the results of the model appropriately, and for them to engage in shaping the technology [16].

Considering AIs to have some level of agency has been an element of human-computer interaction work regarding interactions with AIs for a long time [14]. From the early ‘direct manipulation’ systems [18], agency has developed significantly with the ‘rise of machine agency’ and substantially increased to a level which machines can act and influence, exerting their own agency [19]. This machine agency is not necessarily equal to human agency—there exist good reasons for distinguishing the moral agency of humans from that of machines including that we might otherwise be abdicating human responsibility [20]. However, with assistants such as Apple Siri, Amazon Alexa and Google Assistant, it is increasingly evident that we can interact with and consider AIs with some level of agency, especially in the context of requesting an explanation.

The increasingly complex delivery of healthcare requires strong teamwork. There is a move to consider medical professionals like ‘pit crews, not cowboys’ [21]. We should not expect medical professionals to treat patients independently, with scant regard for colleagues or protocols (the ‘cowboy’ metaphor). Rather, they should work as a team of generalists and specialists who each have a part to play in getting the patient back to full health (like the pit crew in Formula 1 racing). Improved teamwork can lead to improved clinical outcomes [22, 23], patient experience [24], and healthcare worker outcomes [23, 25]. Improved clinical outcomes include reduced rates of error and patient mortality [23]. Principal factors of the teamwork include role clarity, mutual trust, and quality exchange of information [22].

Considering an AI in the same terms as a pit-crew member, much like other healthcare professionals, can help address many of the concerns regarding trust and clinical use. The precise role that an AI might fulfil may vary with application. Considering an AI with the same techniques as used with other members of the clinical team may provide new approaches for addressing the concerns regarding its use. The effective application of AI in healthcare requires the same factors as good teamwork, with the role of an AI needing to be well defined, with trust being a motivating factor for explanation, and quality information exchange being synonymous with explanation. Mental models help in understanding and improving teamwork and we explore this and its application to explanation in AI in the next section.

### Mental models

A mental model is a mental representation of the state of the world, especially in a given context. Medically, this might be who is responsible for specific tasks, information about the patient, the plans for treating the patient, and the culture of the unit. Shared mental models help improve teamwork [15, 26] and deliver higher quality care [27, 27, 28].

Team mental models are the shared understanding or representation of the relevant knowledge of a team [29]. Team mental models divide into four categories:

1. The team model which pertains to the knowledge and skills of team members;
2. The team interaction model which pertains to the roles and responsibilities of the individuals;
3. The equipment model which pertains to the equipment and system itself;
4. And the task model which pertains to the actions and procedures relevant to the task at hand [29, 30].

When a team is working on a single task, each member will have their own mental model(s) regarding the task at hand. The overlap of these mental models is the shared mental model [31]. Sharing mental models is thought to improve team performance [31], though complete overlap may not be necessary or optimal [32]. However, a failure to share the mental model within a team may well lead to errors, especially in complex tasks involving coordinated actions within a team [33].

**Fig. 1:**
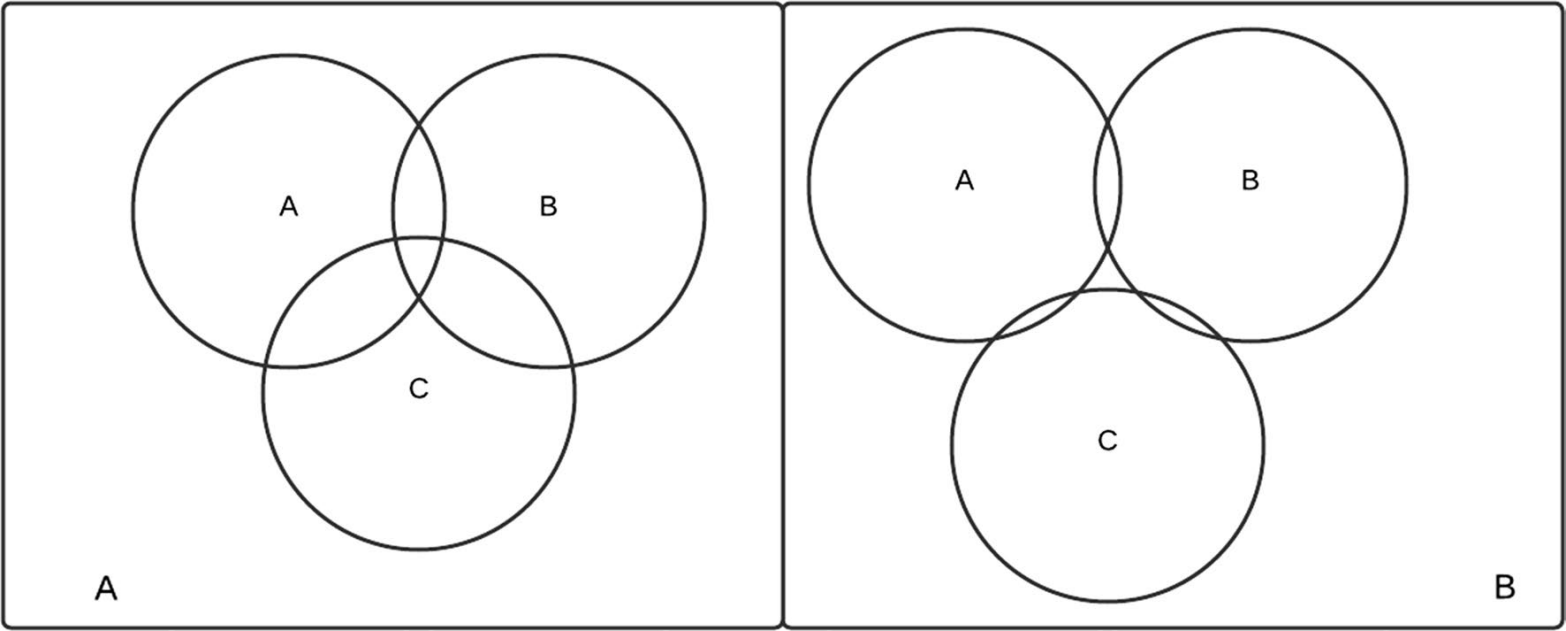
Venn diagrams representing differences in overlapping mental models. **A** Three mental models overlap with a subset being globally understood. **B** Although each pair intersects, there is no globally shared mental model. Inspired by and adapted from [34]

A shared mental model across a team can be understood similar to a Venn diagram, with overlaps between the different mental models (see Fig. 1). There may be a centralized mental model with a common understanding between all individuals within the team. Different combinations within the team might have different overlaps. It is even possible that there exists no part of the mental model shared amongst all team member. Some failures to share the mental model might be of greater risk than others. Two people believing the other is responsible for a critical task could be riskier than each believing they are responsible. In one case, neither takes responsibility, and in the other, both do [34].

Eliciting mental models is the ‘process of inquiry to encourage a person to externalize a mental model’ which yields an expressed mental model [35]. Mental model elicitation is situated (i.e., in the context in which the mental model applies), or non-situated (i.e., away from that context), and can be either oral- or visual-based elicitation [35]. Many techniques exist for eliciting mental models, for example, interviews whilst walking through a relevant area [36], organization of concepts spatially or visually [37], diagrammatic interviews where relationships between concepts are drawn [38] and structured interviews with structures for specific contexts, such as Actors, Resources, Dynamics, and Interaction (ARDI) [39]. Medically, there have been studies examining mental models’ relationship with performance in anaesthesia in the Operating Room (OR) [30, 40], looking at team interactions within surgical teams [41] and trauma teams [28]. Improved sharing of mental models has been identified as a factor to improve the delivery of care [42].

Mental models provide a framework for considering how teams work, and how to improve teamwork. They address questions with regards to roles and how members engage with others in a team, and the research in the area has provided ways to understand the different dynamics of these interactions in a formalised way. Research into mental models has a range of general approaches (e.g., as described by Jones et al. [43]), but that given the range of potential team structures that exist, these approaches are specialised and adapted for specific use within the context of the team in which they are being applied.

### Explainable AI

David Goodstein once asked Richard Feynman to explain why ‘spin one-half particles obey Fermi-Dirac statistics’. Feynman promised to deliver a freshman lecture on the topic. A few days later, he said ‘I couldn’t do it. I couldn’t reduce it to the freshman level. That means we don’t really understand it.’[44]. Although talking about physics models, this suggests a general heuristic regarding explanation: you understand the model if you can explain it at a freshman level. There is a wide range of definitions for explanation that have been proposed [6–8, 12]. In this section, we present two definitions, particularly from Guidotti et al. [6] and Doshi-Velez and Kim [7]. We then present a critique of these approaches, and the computer-science-oriented approach more generally.

#### Guidotti et al. [6]

Guidotti et al. [6] provide a comprehensive review of existing approaches to explanation in machine learning to create a practical definition. They provide the following series of definitions:

- ‘An explanation is an ‘interface’ between humans and a decision-maker that is at the same time both an accurate proxy of the decision-maker and comprehensible to humans.’
- ‘We refer to interpretability also with the name comprehensibility.’
- ‘Interpretability [...] provide[s] meaning in understandable terms.’

They then provide a taxonomy of explanations, categorizing them as model explanations (explaining the logic of a classifier); outcome explanations (explaining the reasons for a given decision); or model inspection (explaining how the model behaves internally), before providing details of the taxonomy with examples. Within this taxonomy, they distinguish between local and global explanations. Local explanations relate to a single prediction or decision whereas global explanations cover the entirety of the model and all possible predictions. Globally explainable models are a superset of locally explainable models—if you are able to explain all predictions, you can explain a single one. Global explanation is therefore a harder problem to solve, especially as many local techniques do not apply globally.

This series of definitions contains two important components, the definition of the agents involved as the humans and the decision-maker, and the claim that an explanation is a proxy that is comprehensible/interpretable/understandable. This definition is characteristic of many approaches in explainable AI. It leans heavily on various terms, particularly creating a chained definition via the synonymous terms ‘interpretability’, ‘comprehensibility’ and ‘understandable’. However, it doesn’t go so far as to formally define those terms. The context is very general (as to who/what are the humans and decision-makers) and the practical steps are highly specified. The idea is that a given model plus an explanatory method should hold generally. For example, LORE [6] which creates a local explanation for any predictive model, should be applicable to any model for any predictive purpose and be equally as applicable. Whether or not the model to be explained is a neural network or a support vector machine, and whether or not it is a medical or a house-prices application, LORE is equally applicable, allowing for a wide range of use cases.

#### Doshi-Velez and Kim [7]

Doshi-Velez and Kim [7] also attempted to define explainability and make the approach more rigorous. They identify two approaches to explanation in the literature: the idea of explanation via a proxy (as per [6]); and the idea of interpretability in the context of an application. They characterise the first in the following way: ‘if the system is useful in either a practical application or a simplified version of it, then it must be somehow interpretable’. They characterise the second as ‘interpretability via a quantifiable proxy’—representing the model using another that is claimed to be interpretable. These two approaches they reject as being weak and instead propose that ‘interpretability [is defined] as the ability to explain or to present in understandable terms to a human’. Similar to Guidotti et al., they do not formalise the definitions of ‘explain’ or ‘understandable’. They look at the evaluation in the context of the task at hand ‘such as whether [the explanation] results in better identification of errors, new facts or less discrimination’. A model that assists in a task is interpretable if the human is able to perform the task better, such as a student doing better in a test.

Doshi-Velez and Kim identify an important element for research moving forward—that the evaluation should match the claim, with the example of reliability claims being poorly supported by accuracy-based evaluations. This puts forward a new perspective compared to other researchers, especially where the methods are being undertaken on specific, real-world tasks. Previous research has made many claims on various quantitative metrics of explanations, such as complexity or size of the models [6]. These are measures that are relatively easy to compute as part of an automated set of experiments in code, but not necessarily reflective of the experience of the individuals who receive the explanations. This is perhaps unsurprising for computer-science research, though does indicate a possible limitation of the existing approaches when considering their application in applied contexts.

There is also a commonly-made claim regarding explanation of computational models: there exist models that are inherently comprehensible, such as rule-based classifiers, decision trees, decision sets, and rational functions [6, 7, 45]. A number of approaches, such as LIME and LORE [6, 11] approximate the target model using various inherently explainable models without necessarily examining this claim in more depth with regards to their definitions. Applying Guidotti et al.’s definition to a regression model does mean that it is explainable—the interface is the mathematical representation of the model, which can be presented to humans, and is understandable for anyone with appropriate training in statistics or similar disciplines. In general, it meets all of the criteria; but one can find and construct counterexamples that would not be considered explainable by many. For example, if one had a regression model with 1,000 inputs, with hundreds of interaction terms, and many computed features, then it might well be considered unintelligible (unexplainable, uninterpretable, incomprehensible). Moreover, for anyone without appropriate training, even a simple regression is not necessarily understandable—p-scores and confidence intervals are notoriously unintuitive. For predictive models in medicine, such as the EuroSCORE II [46], the models typically have a small number of factors and few interactions. These are interpreted by medical professionals with appropriate training. In this context, it could be reasonable to consider the model explainable - it does meet the requirement of ‘understandable to a human’ as per Markus et al. [12]. However, many patients would not find the model intelligible without the interpretative assistance of their doctor.

The computer science claims for explanation follow a theme of generalized context and specified implementation. Recent movements are towards evaluation being more application-oriented, but without a framework for structuring this. Even broadly-accepted assumptions regarding which models are explainable do not necessarily hold when applied in practical contexts. The methods for evaluation are often quantitative and oriented towards features that are easily measurable within computer science labs. The result is that despite all of this work, the use of models in practice such as clinical decision support systems have not had wide-scale adoption [3]. The next section examines a change in approach that might help resolve these conflicts.

### Mental models for explainable artificial intelligence

Mental models are commonly used within the contexts of teams, which could be considered multi-agent contexts from a computer science perspective. They are a representation that facilitates the combined use of knowledge and skills to achieve tasks together. This is equally applicable to the use of a computational model or AI system. In some contexts, it is a collaborative effort for the user and the model to jointly achieve a task, or a task might be delegated to the model for it to achieve independently. There is a parallel between the elicitation of a mental model of a team member and the creation of an explanation from an AI agent. In the first, we are concerned around understanding how a team member is thinking in order to improve teamwork and reduce errors. In the second, we are concerned with how an AI reaches a prediction or decision in order to function better at the task at hand, addressing potential concerns such as bias and inaccuracy. It is with this lens that we aim to fit computational models into the mental-model framework—by considering them as agents within the team whose mental model must be shared.

If this is to be applied to computational models and AI, then rather than developing general methods out of the context of the practical application, explainable AI research ought to focus on explanations that make sense within their intended context. The context is similar to that of the mental model: who is the audience of the explanation, what is the language that is being used to communicate, and what is the purpose of the model. This is not entirely dissimilar to the previous definitions but does require that an explanation have these three elements defined (audience, language, and purpose) in order to guide its evaluation (as per the recommendations of Doshi-Velez and Kim [7]). Rather than being a general ‘interface’, an explanation is a representation in a language that is appropriate for a specific audience in a way that is relevant to the task at hand.

On that basis, we present the following series of definitions for the explanation of models that are equally applicable to mental and computational models.

1. A *model* is a simplified representation of a thing that enhances the ability to understand, predict and possibly control. [47]
2. An *explanation of a model* is an expression or reformulation of the model in a different medium that can be shared with others.
3. An *accurate expression* of a model is one that would allow an accurate reproduction of the results of the model (allowing for some additional computational or logical work).
4. The model and explanation have a purpose, an audience, and a language. The purpose, audience and language together form the *context* of the explanation.
  - The *purpose* is the goal for which the model and explanation were created.
  - The *audience* are those people for whom the model was built and who will receive the explanation.
  - The *language* is the form of expression that is used to create the explanation.
5. A *good explanation* is an accurate expression of the model in language that is understandable by the audience that helps in achieving the purpose.
6. A model is *explainable* if a good explanation of the model can be created.

The clarification of *context* is the primary change in approach between the combined work of Guidotti et al. [6] and Doshi-Velez and Kim [7] and this definition which reflects the ideology of mental models. Rather than defining the metrics for the quality of explanation in a general way and then applying them in context, here we propose defining the metrics in context from the beginning and look for ways of eliciting those explanations to meet the goals. This is aligned with the mental-models approach where the interaction between team-members is being considered within context. This change of approach does not invalidate previous research or methods but requires researchers to consider the practicalities of the use case at hand. It particularly establishes the three most important factors to consider—the purpose, audience, and language.

A clearly stated *purpose* is necessary to evaluate an explanation. It may be useful to decompose any given purpose into sub-goals, having one high-level purpose and separate, targeted goals for each of the model and explanation. For example, if the purpose of developing an AI is to improve patient outcomes for patients at risk of cardiovascular disease in primary care, then the goal of the model could be to create better risk score predictions, and the goal of the explanation could be to allow general practitioners to better communicate the risk factors and possible interventions to patients. The purpose might vary even for the same model and this applies equally to non-computational contexts. A professor delivering lectures on structural engineering principles explains a range of concepts. She might have a range of goals. On one hand, she might be focused on the pass-rate of the course, allowing students to learn. She might also be considering how well the students can use the content after the exams, or even how her lectures might inspire some students to undertake post-graduate study with her. The evaluation of the professor’s lectures would vary depending on which of these purposes was considered, with candidate measures such as pass rates in examinations, total failure rate of bridges built in the next 50 years, and retention of students for post-graduate studies.

An explanation requires an *audience*. As different audiences have different levels of assumed knowledge, language, and familiarity with presentation style, evaluating an explanation against a specific audience is necessary. The same explanation may be more or less appropriate for different audiences. For example, a good explanation of an AI used to predict cardiovascular disease risk in order to improve health outcomes for patients changes if it is the physician, patient, or both who is the audience. Similarly, a professor’s lectures may be considered well-explained to a post-graduate audience but inappropriate for high-school students.

The appropriate *language* to explain things differs depending on the audience. The language of an explanation may be generic or domain-specific. Here, language is used broadly to also capture other representations, such as charts, formulae, and physical representations. The choice of language is important and should be aligned with the audience, rather than the model or those creating the model and the explanation, in the same way that the language used in a lecture changes for high-school courses compared to post-graduate classes on the same topic. Although a model may have a natural representation, such as a mathematical formula, or a series of coefficients and confidence intervals, the appropriate language for a given audience might be a plot of the boundaries, or even a simplified, natural-language description of the relationship between factors and outcomes.

Requiring the full *context* in order to evaluate an explanation provides the terms of reference for any evaluation and provides a minimum set of dimensions to qualify any generic methods. For example, we can examine the claim that a regression model is inherently explainable. The language of the explanation is typically the formula (e.g., $y=b_0+b_1 x_1+b_2 x_2+\ldots +b_n x_n$), with the $b_n$ coefficients and description of the $x_n$ factors, along with confidence intervals or some similar uncertainty representation. The audience is varied, but we can make a broad claim that this would be appropriate for anyone with some training in statistical models, especially regression models, and who is familiar with linear relationships. They must also be familiar with what the $x_n$ factors mean, which may be more challenging if any are computed features. Provided those conditions are met, many purposes would be satisfied with a good explanation (e.g., understanding what factors have a linear relationship with the outcome variable). A patient wanting to understand why he is at high-risk for cardiovascular disease during a consultation with his physician, however, may not have the relevant statistical training nor understanding of the specific factors. If the explanation is to explain to him what is happening with his health so that he can make positive lifestyle changes, then the regression model is likely an inadequate explanation. It is only via an intermediary (e.g., a clinician) that such an explanation would be considered good, but this contradicts the idea of the inherent explainability of the model.

The *accurate expression* is important to distinguish from the accuracy of the model. The explanation is a representation of the model, and the model is a representation of the data. The definition above requires that the explanation be accurate to the model, and allows that the model may not be accurate to the data (in fact, one may identify from an explanation that the model has clear inaccuracies with respect to modelling the data). This can also be described as fidelity—‘accurately describing the task model’ [12]. However, the explanation may not necessarily be as precise as the model, while still retaining the accuracy. Depending on the context, this may be more or less of a problem, and we explore this below in Case study 1.

### Case studies

The following are a set of three short case studies examining different models (computational and mental) and how this method of explanation might apply to them. These case studies are presented as worked examples of the new definition, and show how the definition can apply to existing work in the literature. These are chosen to be an interesting range of cases focused on healthcare, demonstrating the applicability of the definition in a range of contexts.

#### Case study 1: PREDICT and the New Zealand Primary Care Handbook

**Fig. 2:**
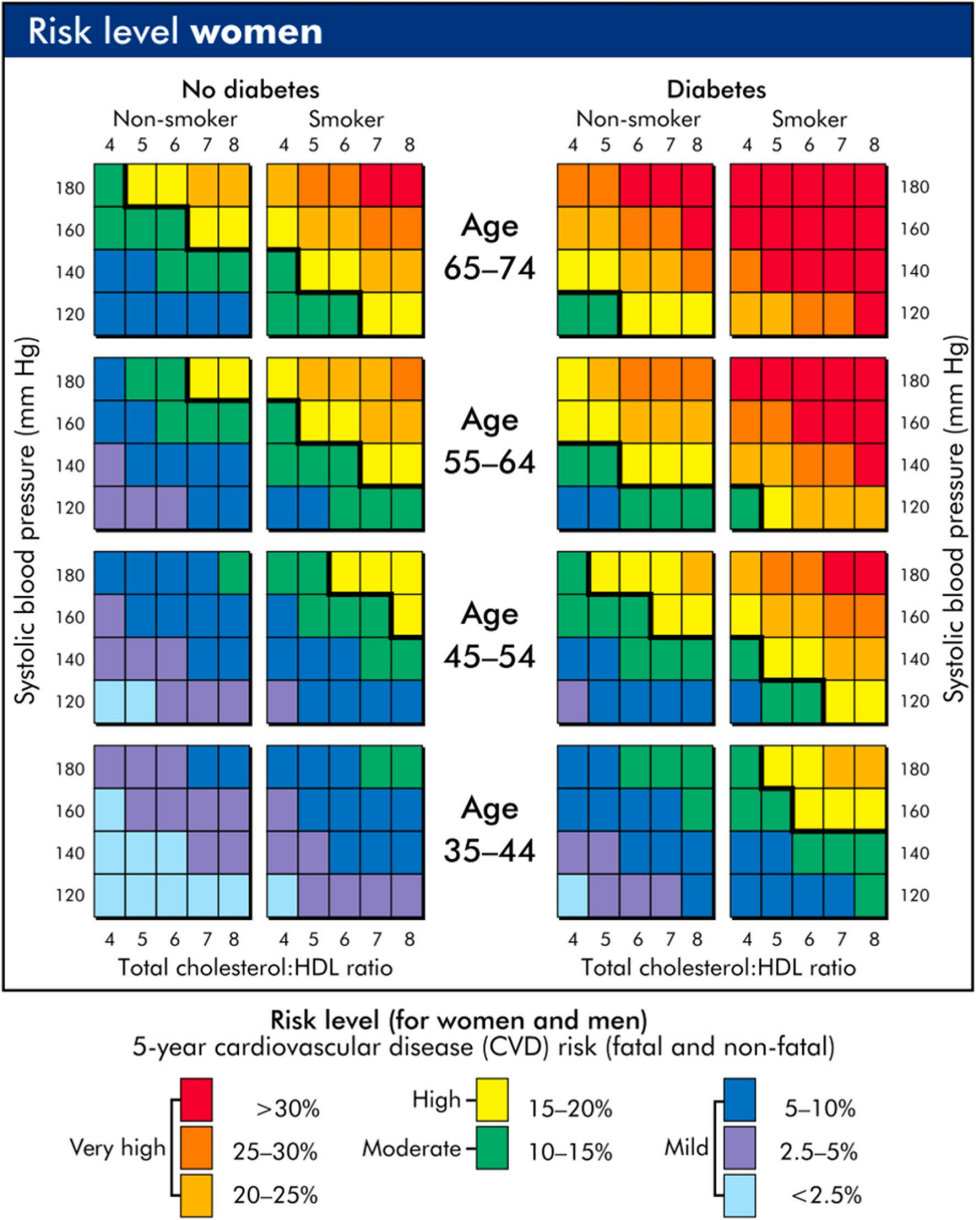
Example of charts referencing cardiovascular disease risk prediction models for primary-care use in New Zealand from Fig. 1 of [52]

The PREDICT clinical decision support tool is a web-based system that integrates with general practice electronic health records for cardiovascular disease (CVD) risk assessment and data collection [48]. PREDICT was built on the New Zealand guidelines for managing cardiovascular risk [49] and includes a risk-prediction model based on the Framingham model [50]. This has been revised for the New Zealand population, most recently in 2018 [51]. This related work was the basis for a set of risk-assessment charts in the New Zealand Primary Care Handbook [52]. These charts (see Fig. 2) are a summary of the model which used a combination of demographic and test results, including age, sex, smoking status, diabetes status, blood pressure, and cholesterol. These charts provided a quick lookup of the risk level (categorised in various bands ranging from mild to very high using colour coded grids) of a patient according to the risk-prediction model and could well be considered an explanation of the model which could easily be used by the general practitioner or be presented to a patient.

Applying the above definitions, we have the risk-prediction model, for which the handbook contains the charts that are the explanation. These are accurate representations to the level of granularity that they specify (risk bands such as 2.5–5%). The purpose of the model is to facilitate the delivery of care for patients at risk of cardiovascular disease, both to estimate the risk and to guide the treatment. The audience of the explanation is the general practitioner and the patients, and the language used is graphical charts. An appropriate evaluation of how good this explanation is could be done by a sociological study of the uptake and comprehension, and an epidemiological or randomised controlled trial regarding the improvement in outcomes for those patients for whom this was used.

#### Case study 2: LYNA

The Lymph Node Assistant (LYNA) [53, 54] is a convolutional neural network that is used to help detect metastases in gigapixel microscopy images. It analyses the images, identifies areas of potential metastases and highlights them on screen for further review by pathologists. The LYNA model provides highlighted areas of the scan to help detect metastases. These are accurate representations that highlight the areas it has detected. The audience is the pathologists, and the language is highlighted or encircled regions on the scans. What is not so explicit within the research is whether or not the goal is simply diagnosis and identification, or whether or not there is an element of understanding why a given part of the image relating to the metastasis is classified as cancerous compared to other parts, and what are the features that are of importance. Depending on which of those two goals is most important, the evaluation might be different. In the first case, a randomized controlled trial of the use of LYNA in context might be more appropriate, and in the second, an analysis of whether or not new features and hypotheses could be generated with regards to metastatic cancer formation that could be tested by other means.

#### Case study 3: operating room mental models

**Fig. 3:**
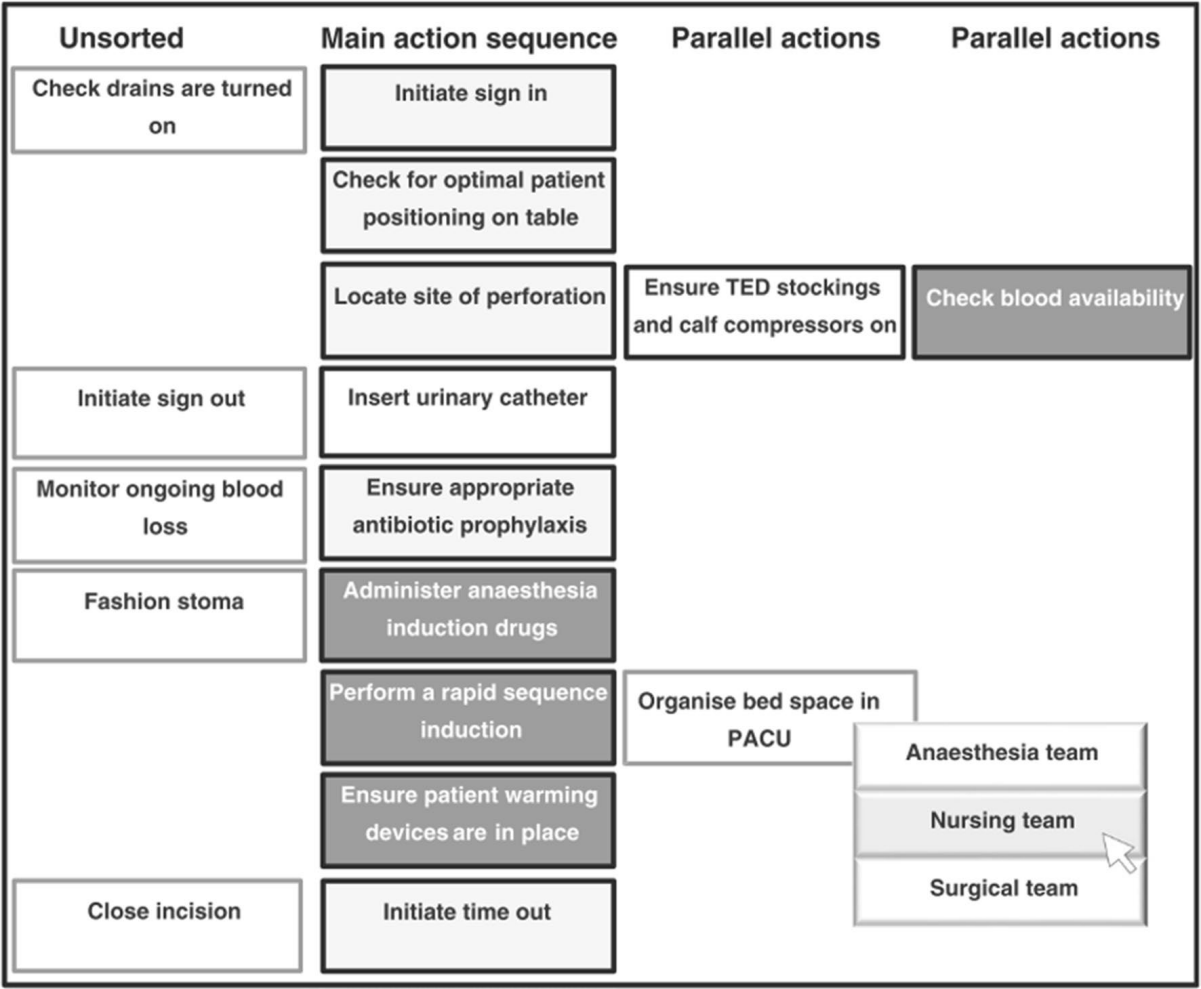
Card sorting algorithm in progress from Fig. 1 of [41]

Nakarada-Kordic et al. [41] captured the mental models of operating room teams comprising of nurses, anaesthetists, and surgeons, with regards to what order tasks should be done in and whose responsibility they were. These were analysed for similarity within the team, with a view to improving the delivery of care for patients and a reduction of errors. The models were the mental models of task allocation of the surgical team for which the explanation was the results of a card-sort elicitation (see Fig. 3). The purpose of the model is to deliver high-quality, error-free surgical care to patients. The audience of the explanation is the research team and the surgical team themselves, and the language is the list of tasks in order with whose responsibility it is next to it. An appropriate evaluation of the explanation is whether or not it allowed for effective analysis of the mental models and allowed for the identification of discrepancies, overlaps, and potentially dangerous misunderstandings between different teams.

## Discussion

The definition of explanation provided in this paper implies that a change in approach would be necessary for future research in explainable AI. Previous research has worked with abstracted deployment contexts, with an implicit idea that a technique once developed can and should be able to be applied broadly. Although this approach has merit and yields results like convolutional neural networks—a technique that can be applied successfully in a large number of tasks—it is not a method that can be applied as successfully for explanations. This idea already has a parallel within the explanatory methods, namely the idea of global vs local explanations. Although this idea has been applied to global or local regions of a given model, it is relevant to explaining models. Rather than a method for explaining all neural networks irrespective of context which might be approximate at a local scale, the idea is to explain specific neural networks that take into account the locality in terms of the context in which an explanation will be delivered.

Arrieta et al. [55] stated that an audience should be given to define explainability, and also highlighted the need of a metric to evaluate how well a model meets the definition. The underlying conflict here is precisely what we have looked to address. If one desires a common metric to compare multiple models, but presupposes a specified audience, then either the choice of audience doesn’t impact the explainability or the metric must depend on the audience, not the model. We agree that any explainability metric must be audience-dependent, and moreover context-dependent. Just as Feynman’s explanation presupposed freshmen students, rather than high-school or post-graduate students, so too must the explanation of a model for a doctor be evaluated differently than one targeted for a patient. This may mean that a common metric is not possible, but that does not mean that a common approach is not viable. This will likely require increased work on the part of XAI researchers to clarify and work with the stakeholders for whom they are targeting their explanations, but as well this should result in much more useful and better-justified explanations as a result.

The use of this definition does not require that a pre-hoc definition of context has been given, but can be used post-hoc to qualify the explainability of models that already exist. For example, if we apply this definition to logistic regression models, we can challenge and qualify the claim that they are inherently explainable. Many regressions are reasonably considered explainable if we assume standard statistical outputs as the language, and an audience with statistical training. This does, however, restrict the scope of the explainability substantially from being inherently explainable to anyone in the world. Even then, for the sake of argument, we can consider a regression with 10,000 terms, with many complex interactions. The output from a statistical package is likely not understandable even for those with statistical training - further restrictions of the audience, or a refinement of the language might be required as the complexity increases. Additionally, even simpler models in more complex domains might require other restrictions on the audience. For example, survival models in epidemiological contexts might require specific training and additional domain knowledge to meet the definition of explainable. Providing precise boundaries of existing techniques will allow us to better use the them, and to identify gaps where we may not have realised they existed.

It may also be necessary to consider the range of purposes for which different approaches provide good explanations. For example, proxy methods may not be appropriate for addressing the concerns of bias in AIs. If we define the purpose of the explanation as being ‘to assuage the concerns of the general public about the use of the model’, then the argument can be made that creating an explanation on the basis of a similar but distinct model may not be sufficient to assuage the concerns. It is not necessarily the role of the researchers to make that determination (except possibly via sociological research), and what is acceptable may vary widely between locations and cultures. We may also come to different conclusions if we define the purpose as being ‘to prove that there exists no discriminatory effect on the basis of racial or ethnic origin, political opinion, religion or beliefs, trade union membership, genetic or health status or sexual orientation in the model’ (adapted from [2]). The level of proof required for a regulatory body may be that the explanation is not on the basis of an approximation and should be directly based on the model of concern. Comparatively, if we are looking to generate hypotheses for future research, the results of which can be verified via other means, then the same level of rigour may not be needed and a wider range of techniques can be used.

An area that needs significant additional work is the examination of the language of explanations that are provided. Many explanations are created and evaluated by computer scientists, and things like rules in decision trees and the coefficients of numerical models are a language that is most familiar to people from numerical disciplines. Engaging a wider range of stakeholders in the context where a model might be applied to help drive what could be a reasonable explanation will help broaden and enrich the languages that we have to explain models. It may mean that methods for explanation become less amenable to statistical analysis, and this might mean that other forms of evaluation must be considered, with either measurable secondary effects (e.g., model uptake, improved delivery of care) or qualitative analyses being necessary.

## Conclusions

The motivation behind explanations of AI and computational models are grounded in practical applications, especially in sensitive contexts. In the same contexts, mental models and the elicitation thereof have been useful in improving teamwork. By considering an AI as part of the team, we can be motivated by the mental models approach in defining and generating explanations for the AI. In contrast to previous definitions, our definition focuses on the context in which the AI is used, and requires the specification of the language, audience, and purpose of the model. By contextualising the AI, explicit engagement with the audience and other stakeholders becomes a necessary part of the development of explanations. Further research in this area should be on the basis of this stakeholder engagement, and the work presented here provides a framework for that work.

## Data Availability

Not applicable.

## Abbreviations

AI: Artificial Intelligence
LYNA: Lymph Node Assistant
LORE: Local Rule-based Explanations
GDPR: General Data Protection Regulation
ARDI: Actors, Resources, Dynamics, and Interaction
OR: Operating Room
LIME: Locally Interpretable Model-agnostic Explanations
